# Bosentan monohydrate

**DOI:** 10.1107/S1600536812048969

**Published:** 2012-12-05

**Authors:** Manpreet Kaur, Jerry P. Jasinski, Amanda C. Keeley, H. S. Yathirajan, Richard Betz, Thomas Gerber, Ray J. Butcher

**Affiliations:** a Department of Studies in Chemistry, University of Mysore, Manasagangotri, Mysore 570 006, India; b Department of Chemistry, Keene State College, 229 Main Street, Keene, NH 03435-2001, USA; c Nelson Mandela Metropolitan University, Summerstrand Campus, Department of Chemistry, University Way, Summerstrand, PP Box 77000, Port Elizabeth 6031, South Africa; d Department of Chemistry, Howard University, 525 College Street NW, Washington, DC 20059, USA

## Abstract

In the title compound, C_27_H_29_N_5_O_6_S·H_2_O {systematic name: 4-*tert*-butyl-*N*-[6-(2-hy­droxy­eth­oxy)-5-(2-meth­oxy­phen­oxy)-2-(pyrimidin-2-yl)pyrimidin-4-yl]benzene-1-sulfonamide monohydrate], the dihedral angle between the mean planes of the pyrimidine rings is 27.0 (1)°. The dihedral angle between the mean planes of the benzene rings is 47.7 (8)°, forming a U-shaped channel around the chain of twisted pyrimidine rings. The crystal packing is stabilized by O—H⋯O, O—H⋯N and N—H⋯O hydrogen bonds with a single water mol­ecule, and weak O—H⋯N inter­molecular inter­actions between the hy­droxy group and one of the pyrimidine rings producing an two-dimensional supra­molecular array in the *bc* plane. The title compound studied was a merohedral twin with the major component being approximately 57%.

## Related literature

For reviews of bosentan in the management of pulmonary arterial hypertension and systemic sclerosis, see: Gabbay *et al.* (2007 ▶); Kumar *et al.* (2011 ▶); Oldfield & Lyseng-Williamson (2006 ▶). For related structures, see: Singh *et al.* (1985 ▶); El-Ghamry *et al.* (2008 ▶); Kant *et al.* (2012 ▶).
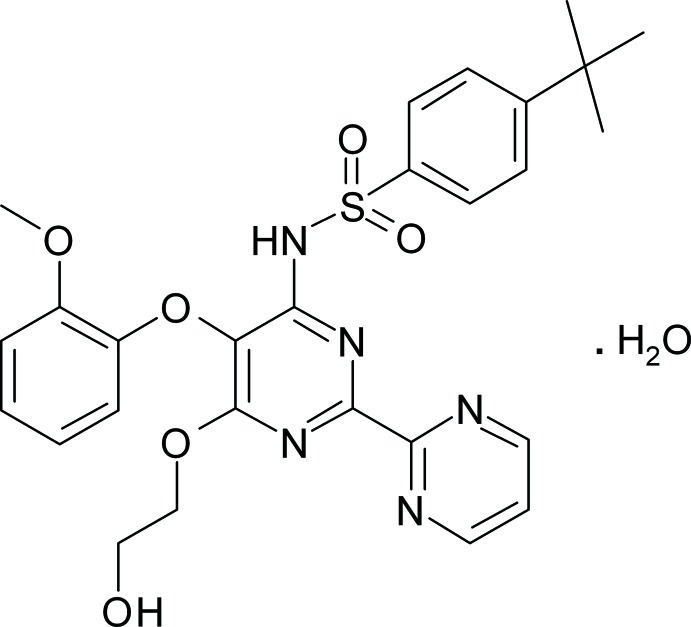


## Experimental

### 

#### Crystal data

C_27_H_29_N_5_O_6_S·H_2_O
*M* *_r_* = 569.63
Monoclinic, 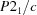

*a* = 12.3393 (4) Å
*b* = 15.1238 (6) Å
*c* = 14.6988 (4) Å
β = 95.037 (3)°
*V* = 2732.46 (16) Å^3^
*Z* = 4
Cu *K*α radiation
μ = 1.52 mm^−1^
*T* = 123 K
0.79 × 0.43 × 0.22 mm

#### Data collection

Agilent Xcalibur (Ruby, Gemini) diffractometer
Absorption correction: analytical (*CrysAlis RED*; Agilent, 2012 ▶) *T*_min_ = 0.535, *T*_max_ = 0.766
9563 measured reflections
9563 independent reflections
7477 reflections with *I* > 2σ(*I*)

#### Refinement

*R*[*F* ^2^ > 2σ(*F* ^2^)] = 0.055
*wR*(*F* ^2^) = 0.163
*S* = 1.03
9563 reflections
373 parameters
3 restraints
H atoms treated by a mixture of independent and constrained refinement
Δρ_max_ = 0.44 e Å^−3^
Δρ_min_ = −0.77 e Å^−3^

### 

Data collection: *CrysAlis PRO* (Agilent, 2012 ▶); cell refinement: *CrysAlis PRO*; data reduction: *CrysAlis RED* (Agilent, 2012 ▶); program(s) used to solve structure: *SHELXS97* (Sheldrick, 2008 ▶); program(s) used to refine structure: *SHELXL97* (Sheldrick, 2008 ▶); molecular graphics: *SHELXTL* (Sheldrick, 2008 ▶); software used to prepare material for publication: *SHELXTL*.

## Supplementary Material

Crystal structure: contains datablock(s) global, I. DOI: 10.1107/S1600536812048969/tk5176sup1.cif

Structure factors: contains datablock(s) I. DOI: 10.1107/S1600536812048969/tk5176Isup2.hkl

Supplementary material file. DOI: 10.1107/S1600536812048969/tk5176Isup3.cml

Additional supplementary materials:  crystallographic information; 3D view; checkCIF report

## Figures and Tables

**Table 1:** Hydrogen-bond geometry (Å, °)

*D*—H⋯*A*	*D*—H	H⋯*A*	*D*⋯*A*	*D*—H⋯*A*
O6—H6⋯N3^i^	0.84	2.51	3.317 (2)	162
O6—H6⋯N4^i^	0.84	2.60	3.141 (2)	124
O1*W*—H1*W*⋯O4	0.80 (2)	2.30 (2)	3.013 (2)	150 (3)
O1*W*—H2*W*⋯N4^ii^	0.81 (2)	2.07 (2)	2.873 (2)	174 (3)
N1—H1*A*⋯O1*W*	0.88	1.87	2.721 (2)	163

Symmetry codes: (i) 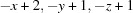; (ii) 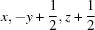.

**Fig. 1.**
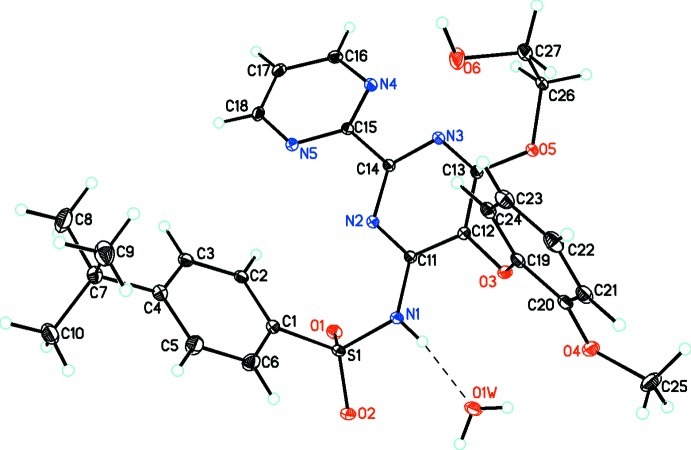
Molecular structure of the title compound showing the atom labeling scheme and 30% probability displacement ellipsoids. The dashed indicates N1—H1···O1W hydrogen bonding within the asymmetric unit.

**Fig. 2.**
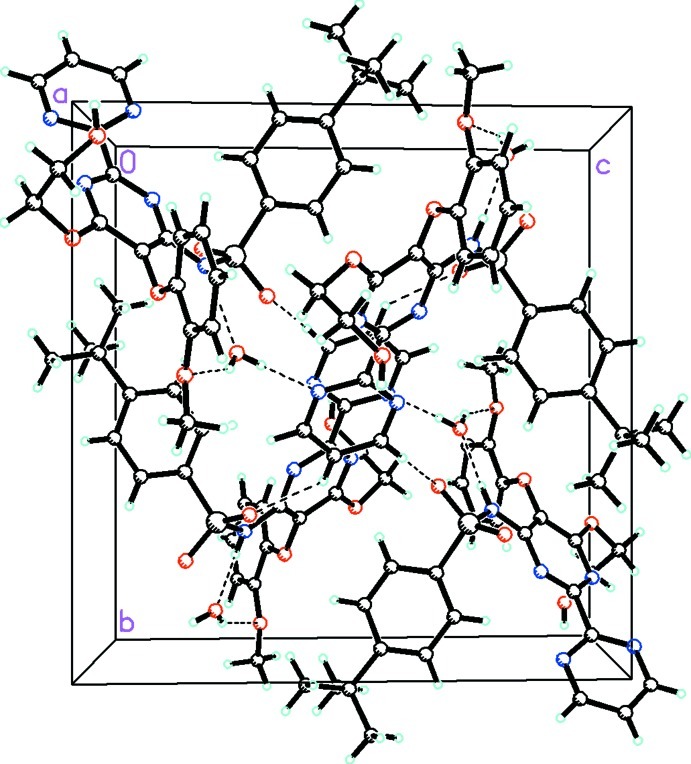
Packing diagram of the title compound viewed along the a axis. Dashed lines indicate O—H···O, O—H···N and N—H···O hydrogen bonds with a single water molecule and weak O—H···N intermolecular interactions between the hydroxy group and one of the nearby pyrimidine rings. The remaining H atoms have been removed for clarity.

## supplementary crystallographic information

### Comment

Bosentan (chemically, 4-tert-butyl-N-[6-(2-hydroxyethoxy)-5-(2-methoxyphenoxy) -2-(pyrimidin-2-yl)pyrimidin-4-yl]benzene-1-sulfonamide) is a dual endothelin receptor antagonist used in the treatment of pulmonary artery hypertension (PAH). Bosentan is used to treat pulmonary hypertension by blocking the action of endothelin molecules that would otherwise promote narrowing of the blood vessels and lead to high blood pressure. A review of bosentan in the management of pulmonary arterial hypertension is published (Gabbay *et al.*, 2007). Another review on the use of bosentan in pulmonary arterial hypertension and systemic sclerosis is also published (Oldfield & Lyseng-Williamson, 2006). A spectrophotometric method for the determination of bosentan monohydrate in bulk and pharmaceutical dosage forms is reported (Kumar *et al.*, 2011). The crystal structures of some related compounds, *viz*., N1-(2,6-dimethyl-4-pyrimidinyl)sulphanilamide (Singh *et al.*, 1985), 4-[(3-formyl-4-hydroxyphenyl)diazenyl]-N-(pyrimidin-2-yl)benzenesulfonamide (El-Ghamry *et al.*, 2008) and N-(2-{[5-bromo-2-(morpholin-4-yl)pyrimidin-4-yl]sulfanyl}-4-methoxyphenyl)- 4-methyl benzenesulfonamide (Kant *et al.*, 2012), have been reported. In view of the importance of the title compound, (I), the paper reports its crystal structure.

In (I), the dihedral angle between the mean planes of the two pyrimidine rings is 27.0 (1)° (Fig. 1). The dihedral angle between the mean planes of the two benzene rings is 47.7 (8)° forming a U-shaped channel around the chain of twisted pyrimidine rings. Crystal packing is stabilized by O—H···O, O—H···N and N—H···O hydrogen bonds with a single water molecule and weak O—H···N intermolecular interactions between the hydroxy group and one of the nearby pyrimidine rings (Fig. 2).

### Experimental

The title compound was obtained as a gift sample from R. L. Fine Chem, Bengaluru, India. X-ray quality crystals were obtained by slow evaporation of ethanol solution (m.p.: 383–385 K).

### Refinement

Water-bound H1W and H2W were located in a Fourier map and restrained (O—H = 0.82 and H1w···H2w = 1.30). All remaining H atoms were placed in their calculated positions and then refined using the riding model with atom—H lengths of 0.93 Å (CH), 0.97 Å (CH_2_), 0.96 Å (CH_3_) and 0.86 Å (NH). U_iso_ were set to 1.19-1.21 (CH, CH_2_), 1.49 (CH_3_) or 1.20 (NH) times *U*_eq_ of the parent atom. The title compound refines as a merohedral twin with BASF = 0.431.

### Crystal data

**Table:**

C_27_H_29_N_5_O_6_S·H_2_O	*F*(000) = 1200
*M**_r_* = 569.63	*D*_x_ = 1.385 Mg m^−^^3^
Monoclinic, *P*2_1_/*c*	Cu *K*α radiation, λ = 1.54184 Å
Hall symbol: -P 2ybc	Cell parameters from 3219 reflections
*a* = 12.3393 (4) Å	θ = 2.9–75.5°
*b* = 15.1238 (6) Å	µ = 1.52 mm^−^^1^
*c* = 14.6988 (4) Å	*T* = 123 K
β = 95.037 (3)°	Prism, colourless
*V* = 2732.46 (16) Å^3^	0.79 × 0.43 × 0.22 mm
*Z* = 4	

### Data collection

**Table:**

Agilent Xcalibur (Ruby, Gemini) diffractometer	9563 independent reflections
Radiation source: Enhance (Cu) X-ray Source	7477 reflections with *I* > 2σ(*I*)
Graphite monochromator	*R*_int_ = 0.000
Detector resolution: 10.5081 pixels mm^-1^	θ_max_ = 76.1°, θ_min_ = 3.6°
ω scans	*h* = −15→14
Absorption correction: analytical (*CrysAlis PRO*; Agilent, 2012)	*k* = −18→18
*T*_min_ = 0.535, *T*_max_ = 0.766	*l* = −18→18
9563 measured reflections	

### Refinement

**Table:**

Refinement on *F*^2^	Primary atom site location: structure-invariant direct methods
Least-squares matrix: full	Secondary atom site location: difference Fourier map
*R*[*F*^2^ > 2σ(*F*^2^)] = 0.055	Hydrogen site location: inferred from neighbouring sites
*wR*(*F*^2^) = 0.163	H atoms treated by a mixture of independent and constrained refinement
*S* = 1.03	*w* = 1/[σ^2^(*F*_o_^2^) + (0.1185*P*)^2^] where *P* = (*F*_o_^2^ + 2*F*_c_^2^)/3
9563 reflections	(Δ/σ)_max_ < 0.001
373 parameters	Δρ_max_ = 0.44 e Å^−^^3^
3 restraints	Δρ_min_ = −0.77 e Å^−^^3^

### Special details

**Table:**

Geometry. All esds (except the esd in the dihedral angle between two l.s. planes) are estimated using the full covariance matrix. The cell esds are taken into account individually in the estimation of esds in distances, angles and torsion angles; correlations between esds in cell parameters are only used when they are defined by crystal symmetry. An approximate (isotropic) treatment of cell esds is used for estimating esds involving l.s. planes.
Refinement. Refinement of F^2^ against ALL reflections. The weighted R-factor wR and goodness of fit S are based on F^2^, conventional R-factors R are based on F, with F set to zero for negative F^2^. The threshold expression of F^2^ > 2sigma(F^2^) is used only for calculating R-factors(gt) etc. and is not relevant to the choice of reflections for refinement. R-factors based on F^2^ are statistically about twice as large as those based on F, and R- factors based on ALL data will be even larger.

### Fractional atomic coordinates and isotropic or equivalent isotropic displacement parameters (Å^2^)

**Table:**

	*x*	*y*	*z*	*U*_iso_*/*U*_eq_	
S1	0.61088 (3)	0.25246 (3)	0.77145 (3)	0.02158 (12)	
O1	0.52137 (11)	0.26956 (9)	0.70499 (10)	0.0278 (3)	
O2	0.60422 (11)	0.17933 (9)	0.83307 (9)	0.0286 (3)	
O3	0.90811 (10)	0.17574 (9)	0.64520 (9)	0.0223 (3)	
O4	1.00833 (11)	0.03046 (9)	0.69883 (10)	0.0319 (3)	
O5	0.96944 (10)	0.26749 (8)	0.49814 (9)	0.0211 (3)	
O6	1.06737 (13)	0.43628 (11)	0.54935 (14)	0.0466 (5)	
H6	1.0995	0.4851	0.5474	0.056*	
O1W	0.80006 (14)	0.07096 (12)	0.78315 (12)	0.0431 (4)	
H1W	0.8518 (18)	0.0432 (19)	0.772 (2)	0.065*	
H2W	0.774 (2)	0.050 (2)	0.8265 (16)	0.065*	
N1	0.72198 (13)	0.23031 (11)	0.72150 (11)	0.0240 (3)	
H1A	0.7601	0.1839	0.7413	0.029*	
N2	0.70609 (12)	0.35062 (10)	0.62171 (10)	0.0201 (3)	
N3	0.83084 (11)	0.37054 (10)	0.50764 (10)	0.0193 (3)	
N4	0.69029 (12)	0.49778 (11)	0.43244 (10)	0.0228 (3)	
N5	0.63133 (12)	0.51751 (10)	0.58109 (11)	0.0216 (3)	
C1	0.63930 (15)	0.34843 (13)	0.83661 (13)	0.0234 (4)	
C2	0.61384 (14)	0.43168 (13)	0.80149 (12)	0.0229 (4)	
H2A	0.5839	0.4383	0.7401	0.027*	
C3	0.63269 (15)	0.50540 (13)	0.85735 (13)	0.0254 (4)	
H3A	0.6144	0.5624	0.8337	0.031*	
C4	0.67792 (16)	0.49731 (14)	0.94734 (13)	0.0274 (4)	
C5	0.7032 (2)	0.41272 (16)	0.97988 (15)	0.0376 (5)	
H5A	0.7348	0.4058	1.0407	0.045*	
C6	0.68348 (19)	0.33858 (15)	0.92605 (15)	0.0354 (5)	
H6A	0.7001	0.2814	0.9501	0.042*	
C7	0.70065 (18)	0.57723 (14)	1.01064 (14)	0.0315 (4)	
C8	0.6619 (3)	0.66378 (17)	0.96631 (19)	0.0588 (8)	
H8A	0.5833	0.6609	0.9501	0.088*	
H8B	0.6993	0.6736	0.9110	0.088*	
H8C	0.6783	0.7126	1.0092	0.088*	
C9	0.8239 (2)	0.5843 (2)	1.03558 (19)	0.0508 (7)	
H9A	0.8390	0.6337	1.0779	0.076*	
H9B	0.8607	0.5944	0.9801	0.076*	
H9C	0.8507	0.5292	1.0647	0.076*	
C10	0.6441 (2)	0.56343 (18)	1.09732 (16)	0.0451 (6)	
H10A	0.5657	0.5565	1.0816	0.068*	
H10B	0.6571	0.6148	1.1375	0.068*	
H10C	0.6729	0.5102	1.1288	0.068*	
C11	0.76040 (14)	0.27812 (12)	0.65145 (12)	0.0193 (4)	
C12	0.85494 (14)	0.25075 (12)	0.61345 (12)	0.0194 (3)	
C13	0.88431 (14)	0.29866 (12)	0.53861 (12)	0.0193 (3)	
C14	0.74497 (13)	0.39270 (12)	0.55183 (12)	0.0189 (4)	
C15	0.68504 (13)	0.47494 (12)	0.51997 (12)	0.0192 (3)	
C16	0.63172 (15)	0.56851 (13)	0.40389 (13)	0.0251 (4)	
H16A	0.6321	0.5865	0.3420	0.030*	
C17	0.57088 (15)	0.61632 (12)	0.46094 (14)	0.0249 (4)	
H17A	0.5281	0.6655	0.4395	0.030*	
C18	0.57539 (14)	0.58898 (12)	0.55099 (14)	0.0244 (4)	
H18A	0.5374	0.6222	0.5929	0.029*	
C19	1.01482 (14)	0.18540 (13)	0.68486 (12)	0.0221 (4)	
C20	1.06708 (15)	0.10674 (13)	0.71295 (13)	0.0252 (4)	
C21	1.17366 (16)	0.11063 (15)	0.75333 (13)	0.0301 (4)	
H21A	1.2102	0.0578	0.7729	0.036*	
C22	1.22647 (16)	0.19136 (16)	0.76497 (13)	0.0328 (5)	
H22A	1.2990	0.1936	0.7925	0.039*	
C23	1.17412 (16)	0.26824 (15)	0.73685 (14)	0.0300 (4)	
H23A	1.2105	0.3234	0.7451	0.036*	
C24	1.06718 (15)	0.26529 (13)	0.69604 (12)	0.0245 (4)	
H24A	1.0311	0.3183	0.6762	0.029*	
C25	1.06856 (19)	−0.05017 (16)	0.7061 (2)	0.0476 (7)	
H25A	1.0213	−0.0994	0.6847	0.071*	
H25B	1.0954	−0.0601	0.7701	0.071*	
H25C	1.1303	−0.0464	0.6687	0.071*	
C26	1.02013 (15)	0.32487 (12)	0.43522 (12)	0.0233 (4)	
H26A	0.9652	0.3658	0.4056	0.028*	
H26B	1.0502	0.2890	0.3870	0.028*	
C27	1.10957 (16)	0.37666 (15)	0.48616 (15)	0.0323 (5)	
H27A	1.1613	0.3355	0.5195	0.039*	
H27B	1.1498	0.4104	0.4422	0.039*	

### Atomic displacement parameters (Å^2^)

**Table:**

	*U*^11^	*U*^22^	*U*^33^	*U*^12^	*U*^13^	*U*^23^
S1	0.0224 (2)	0.0212 (2)	0.0225 (2)	−0.00108 (16)	0.00979 (17)	0.00304 (17)
O1	0.0249 (6)	0.0310 (7)	0.0278 (7)	−0.0014 (6)	0.0043 (5)	0.0023 (6)
O2	0.0328 (7)	0.0243 (7)	0.0309 (7)	−0.0008 (5)	0.0143 (6)	0.0062 (6)
O3	0.0210 (6)	0.0199 (6)	0.0267 (6)	0.0018 (5)	0.0055 (5)	0.0054 (5)
O4	0.0299 (7)	0.0247 (7)	0.0424 (8)	0.0056 (6)	0.0109 (6)	0.0106 (6)
O5	0.0216 (6)	0.0202 (6)	0.0228 (6)	0.0026 (5)	0.0095 (5)	0.0023 (5)
O6	0.0369 (8)	0.0366 (9)	0.0694 (12)	−0.0127 (7)	0.0228 (8)	−0.0265 (9)
O1W	0.0474 (9)	0.0412 (10)	0.0453 (10)	0.0189 (7)	0.0300 (8)	0.0228 (8)
N1	0.0266 (8)	0.0201 (8)	0.0271 (8)	0.0047 (6)	0.0125 (6)	0.0057 (6)
N2	0.0211 (7)	0.0189 (7)	0.0209 (7)	0.0013 (6)	0.0060 (6)	0.0008 (6)
N3	0.0213 (7)	0.0196 (7)	0.0174 (7)	0.0011 (6)	0.0050 (6)	0.0005 (6)
N4	0.0250 (7)	0.0230 (8)	0.0211 (7)	0.0019 (6)	0.0056 (6)	0.0018 (6)
N5	0.0216 (7)	0.0201 (7)	0.0238 (7)	0.0004 (6)	0.0055 (6)	−0.0004 (6)
C1	0.0250 (8)	0.0220 (9)	0.0244 (9)	0.0010 (7)	0.0082 (7)	0.0005 (7)
C2	0.0248 (8)	0.0247 (9)	0.0200 (8)	0.0008 (7)	0.0070 (7)	0.0040 (7)
C3	0.0276 (9)	0.0258 (9)	0.0238 (9)	0.0020 (7)	0.0075 (7)	0.0051 (8)
C4	0.0318 (10)	0.0272 (10)	0.0238 (9)	0.0008 (8)	0.0053 (8)	−0.0014 (8)
C5	0.0529 (13)	0.0321 (11)	0.0262 (10)	0.0074 (10)	−0.0055 (9)	0.0013 (9)
C6	0.0473 (12)	0.0284 (11)	0.0295 (11)	0.0073 (9)	−0.0014 (9)	0.0056 (9)
C7	0.0409 (11)	0.0285 (11)	0.0255 (10)	0.0000 (9)	0.0046 (8)	−0.0042 (9)
C8	0.105 (2)	0.0305 (13)	0.0398 (14)	0.0115 (14)	−0.0028 (15)	−0.0075 (11)
C9	0.0438 (13)	0.0553 (16)	0.0545 (16)	−0.0129 (12)	0.0121 (12)	−0.0205 (14)
C10	0.0534 (14)	0.0508 (15)	0.0335 (12)	−0.0131 (12)	0.0168 (11)	−0.0133 (11)
C11	0.0226 (8)	0.0179 (8)	0.0183 (8)	−0.0028 (6)	0.0061 (7)	−0.0018 (7)
C12	0.0210 (8)	0.0168 (8)	0.0207 (9)	0.0009 (6)	0.0040 (7)	0.0011 (7)
C13	0.0208 (8)	0.0193 (8)	0.0184 (8)	−0.0015 (6)	0.0053 (6)	−0.0020 (7)
C14	0.0206 (8)	0.0191 (9)	0.0173 (8)	−0.0012 (6)	0.0042 (6)	−0.0010 (7)
C15	0.0184 (7)	0.0183 (8)	0.0213 (8)	−0.0014 (6)	0.0045 (6)	−0.0004 (7)
C16	0.0273 (9)	0.0232 (9)	0.0247 (9)	0.0001 (7)	0.0026 (7)	0.0051 (8)
C17	0.0222 (8)	0.0183 (9)	0.0342 (10)	0.0006 (7)	0.0017 (7)	0.0025 (8)
C18	0.0226 (8)	0.0187 (8)	0.0326 (10)	0.0020 (7)	0.0062 (7)	−0.0028 (8)
C19	0.0212 (8)	0.0287 (10)	0.0175 (8)	0.0046 (7)	0.0071 (7)	0.0034 (7)
C20	0.0258 (9)	0.0285 (10)	0.0230 (9)	0.0068 (7)	0.0110 (7)	0.0074 (8)
C21	0.0293 (9)	0.0401 (12)	0.0220 (9)	0.0123 (8)	0.0078 (8)	0.0075 (8)
C22	0.0267 (10)	0.0491 (13)	0.0226 (9)	0.0051 (9)	0.0027 (8)	0.0023 (9)
C23	0.0277 (9)	0.0385 (12)	0.0239 (10)	−0.0031 (8)	0.0033 (7)	−0.0014 (9)
C24	0.0260 (9)	0.0296 (10)	0.0182 (9)	0.0025 (7)	0.0046 (7)	0.0007 (7)
C25	0.0388 (12)	0.0261 (11)	0.0796 (19)	0.0088 (9)	0.0140 (12)	0.0183 (12)
C26	0.0282 (9)	0.0229 (9)	0.0206 (9)	0.0027 (7)	0.0132 (7)	0.0016 (7)
C27	0.0274 (9)	0.0297 (11)	0.0422 (12)	−0.0023 (8)	0.0163 (9)	−0.0050 (9)

### Geometric parameters (Å, º)

**Table:**

S1—O1	1.4324 (14)	C7—C9	1.537 (3)
S1—O2	1.4365 (14)	C8—H8A	0.9800
S1—N1	1.6455 (15)	C8—H8B	0.9800
S1—C1	1.757 (2)	C8—H8C	0.9800
O3—C12	1.372 (2)	C9—H9A	0.9800
O3—C19	1.400 (2)	C9—H9B	0.9800
O4—C20	1.369 (2)	C9—H9C	0.9800
O4—C25	1.427 (2)	C10—H10A	0.9800
O5—C13	1.338 (2)	C10—H10B	0.9800
O5—C26	1.450 (2)	C10—H10C	0.9800
O6—C27	1.425 (2)	C11—C12	1.400 (2)
O6—H6	0.8400	C12—C13	1.392 (2)
O1W—H1W	0.795 (16)	C14—C15	1.501 (2)
O1W—H2W	0.805 (16)	C16—C17	1.378 (3)
N1—C11	1.376 (2)	C16—H16A	0.9500
N1—H1A	0.8800	C17—C18	1.383 (3)
N2—C14	1.333 (2)	C17—H17A	0.9500
N2—C11	1.338 (2)	C18—H18A	0.9500
N3—C13	1.331 (2)	C19—C24	1.373 (3)
N3—C14	1.333 (2)	C19—C20	1.398 (3)
N4—C16	1.338 (2)	C20—C21	1.396 (3)
N4—C15	1.339 (2)	C21—C22	1.387 (3)
N5—C15	1.329 (2)	C21—H21A	0.9500
N5—C18	1.337 (2)	C22—C23	1.376 (3)
C1—C6	1.386 (3)	C22—H22A	0.9500
C1—C2	1.386 (3)	C23—C24	1.401 (3)
C2—C3	1.392 (3)	C23—H23A	0.9500
C2—H2A	0.9500	C24—H24A	0.9500
C3—C4	1.395 (3)	C25—H25A	0.9800
C3—H3A	0.9500	C25—H25B	0.9800
C4—C5	1.391 (3)	C25—H25C	0.9800
C4—C7	1.536 (3)	C26—C27	1.499 (3)
C5—C6	1.381 (3)	C26—H26A	0.9900
C5—H5A	0.9500	C26—H26B	0.9900
C6—H6A	0.9500	C27—H27A	0.9900
C7—C10	1.520 (3)	C27—H27B	0.9900
C7—C8	1.520 (3)		
			
O1—S1—O2	119.06 (9)	N2—C11—N1	118.65 (15)
O1—S1—N1	110.81 (8)	N2—C11—C12	121.54 (16)
O2—S1—N1	102.75 (8)	N1—C11—C12	119.81 (17)
O1—S1—C1	109.13 (9)	O3—C12—C13	123.34 (16)
O2—S1—C1	108.18 (9)	O3—C12—C11	119.96 (16)
N1—S1—C1	106.09 (9)	C13—C12—C11	116.46 (16)
C12—O3—C19	117.36 (14)	N3—C13—O5	121.38 (15)
C20—O4—C25	116.32 (16)	N3—C13—C12	122.68 (16)
C13—O5—C26	118.20 (14)	O5—C13—C12	115.93 (16)
C27—O6—H6	109.5	N2—C14—N3	127.63 (17)
H1W—O1W—H2W	110 (2)	N2—C14—C15	115.81 (15)
C11—N1—S1	125.60 (13)	N3—C14—C15	116.56 (15)
C11—N1—H1A	117.2	N5—C15—N4	126.42 (17)
S1—N1—H1A	117.2	N5—C15—C14	116.86 (16)
C14—N2—C11	116.05 (15)	N4—C15—C14	116.71 (15)
C13—N3—C14	115.46 (15)	N4—C16—C17	122.46 (18)
C16—N4—C15	115.93 (16)	N4—C16—H16A	118.8
C15—N5—C18	116.21 (16)	C17—C16—H16A	118.8
C6—C1—C2	120.54 (18)	C16—C17—C18	116.55 (17)
C6—C1—S1	118.11 (15)	C16—C17—H17A	121.7
C2—C1—S1	121.29 (15)	C18—C17—H17A	121.7
C1—C2—C3	119.15 (17)	N5—C18—C17	122.32 (17)
C1—C2—H2A	120.4	N5—C18—H18A	118.8
C3—C2—H2A	120.4	C17—C18—H18A	118.8
C2—C3—C4	121.41 (18)	C24—C19—C20	120.89 (18)
C2—C3—H3A	119.3	C24—C19—O3	123.86 (17)
C4—C3—H3A	119.3	C20—C19—O3	115.26 (17)
C5—C4—C3	117.74 (19)	O4—C20—C21	124.53 (18)
C5—C4—C7	119.46 (18)	O4—C20—C19	116.62 (17)
C3—C4—C7	122.81 (19)	C21—C20—C19	118.85 (19)
C6—C5—C4	121.77 (19)	C22—C21—C20	120.29 (19)
C6—C5—H5A	119.1	C22—C21—H21A	119.9
C4—C5—H5A	119.1	C20—C21—H21A	119.9
C5—C6—C1	119.39 (19)	C23—C22—C21	120.24 (19)
C5—C6—H6A	120.3	C23—C22—H22A	119.9
C1—C6—H6A	120.3	C21—C22—H22A	119.9
C10—C7—C8	109.1 (2)	C22—C23—C24	120.1 (2)
C10—C7—C4	109.08 (18)	C22—C23—H23A	120.0
C8—C7—C4	112.58 (18)	C24—C23—H23A	120.0
C10—C7—C9	109.1 (2)	C19—C24—C23	119.65 (19)
C8—C7—C9	108.1 (2)	C19—C24—H24A	120.2
C4—C7—C9	108.85 (18)	C23—C24—H24A	120.2
C7—C8—H8A	109.5	O4—C25—H25A	109.5
C7—C8—H8B	109.5	O4—C25—H25B	109.5
H8A—C8—H8B	109.5	H25A—C25—H25B	109.5
C7—C8—H8C	109.5	O4—C25—H25C	109.5
H8A—C8—H8C	109.5	H25A—C25—H25C	109.5
H8B—C8—H8C	109.5	H25B—C25—H25C	109.5
C7—C9—H9A	109.5	O5—C26—C27	109.51 (15)
C7—C9—H9B	109.5	O5—C26—H26A	109.8
H9A—C9—H9B	109.5	C27—C26—H26A	109.8
C7—C9—H9C	109.5	O5—C26—H26B	109.8
H9A—C9—H9C	109.5	C27—C26—H26B	109.8
H9B—C9—H9C	109.5	H26A—C26—H26B	108.2
C7—C10—H10A	109.5	O6—C27—C26	111.16 (16)
C7—C10—H10B	109.5	O6—C27—H27A	109.4
H10A—C10—H10B	109.5	C26—C27—H27A	109.4
C7—C10—H10C	109.5	O6—C27—H27B	109.4
H10A—C10—H10C	109.5	C26—C27—H27B	109.4
H10B—C10—H10C	109.5	H27A—C27—H27B	108.0
			
O1—S1—N1—C11	47.22 (18)	C26—O5—C13—C12	−165.22 (15)
O2—S1—N1—C11	175.43 (16)	O3—C12—C13—N3	178.87 (16)
C1—S1—N1—C11	−71.10 (18)	C11—C12—C13—N3	4.5 (3)
O1—S1—C1—C6	150.77 (16)	O3—C12—C13—O5	−0.3 (3)
O2—S1—C1—C6	19.86 (18)	C11—C12—C13—O5	−174.61 (16)
N1—S1—C1—C6	−89.80 (17)	C11—N2—C14—N3	0.7 (3)
O1—S1—C1—C2	−26.27 (17)	C11—N2—C14—C15	−178.84 (15)
O2—S1—C1—C2	−157.18 (15)	C13—N3—C14—N2	−1.2 (3)
N1—S1—C1—C2	93.16 (16)	C13—N3—C14—C15	178.36 (15)
C6—C1—C2—C3	−0.3 (3)	C18—N5—C15—N4	1.6 (3)
S1—C1—C2—C3	176.65 (13)	C18—N5—C15—C14	−177.86 (15)
C1—C2—C3—C4	0.9 (3)	C16—N4—C15—N5	−2.9 (3)
C2—C3—C4—C5	−0.4 (3)	C16—N4—C15—C14	176.55 (15)
C2—C3—C4—C7	179.51 (17)	N2—C14—C15—N5	26.1 (2)
C3—C4—C5—C6	−0.7 (3)	N3—C14—C15—N5	−153.49 (16)
C7—C4—C5—C6	179.4 (2)	N2—C14—C15—N4	−153.35 (16)
C4—C5—C6—C1	1.2 (4)	N3—C14—C15—N4	27.0 (2)
C2—C1—C6—C5	−0.7 (3)	C15—N4—C16—C17	1.1 (3)
S1—C1—C6—C5	−177.78 (18)	N4—C16—C17—C18	1.7 (3)
C5—C4—C7—C10	−55.2 (3)	C15—N5—C18—C17	1.6 (3)
C3—C4—C7—C10	124.9 (2)	C16—C17—C18—N5	−3.1 (3)
C5—C4—C7—C8	−176.5 (2)	C12—O3—C19—C24	2.1 (2)
C3—C4—C7—C8	3.7 (3)	C12—O3—C19—C20	−177.61 (15)
C5—C4—C7—C9	63.8 (3)	C25—O4—C20—C21	−15.3 (3)
C3—C4—C7—C9	−116.1 (2)	C25—O4—C20—C19	165.06 (19)
C14—N2—C11—N1	−177.91 (16)	C24—C19—C20—O4	−179.91 (16)
C14—N2—C11—C12	2.5 (3)	O3—C19—C20—O4	−0.2 (2)
S1—N1—C11—N2	2.0 (3)	C24—C19—C20—C21	0.4 (3)
S1—N1—C11—C12	−178.44 (14)	O3—C19—C20—C21	−179.89 (15)
C19—O3—C12—C13	68.0 (2)	O4—C20—C21—C22	−179.81 (18)
C19—O3—C12—C11	−117.88 (18)	C19—C20—C21—C22	−0.2 (3)
N2—C11—C12—O3	−179.54 (16)	C20—C21—C22—C23	0.1 (3)
N1—C11—C12—O3	0.9 (3)	C21—C22—C23—C24	−0.2 (3)
N2—C11—C12—C13	−5.0 (3)	C20—C19—C24—C23	−0.5 (3)
N1—C11—C12—C13	175.48 (16)	O3—C19—C24—C23	179.80 (16)
C14—N3—C13—O5	177.48 (16)	C22—C23—C24—C19	0.4 (3)
C14—N3—C13—C12	−1.6 (3)	C13—O5—C26—C27	90.58 (19)
C26—O5—C13—N3	15.6 (2)	O5—C26—C27—O6	−65.8 (2)

### Hydrogen-bond geometry (Å, º)

**Table:**

*D*—H···*A*	*D*—H	H···*A*	*D*···*A*	*D*—H···*A*
O6—H6···N3^i^	0.84	2.51	3.317 (2)	162
O6—H6···N4^i^	0.84	2.60	3.141 (2)	124
O1*W*—H1*W*···O4	0.80 (2)	2.30 (2)	3.013 (2)	150 (3)
O1*W*—H2*W*···N4^ii^	0.81 (2)	2.07 (2)	2.873 (2)	174 (3)
N1—H1*A*···O1*W*	0.88	1.87	2.721 (2)	163

Symmetry codes: (i) −*x*+2, −*y*+1, −*z*+1; (ii) *x*, −*y*+1/2, *z*+1/2.
